# Transcriptomic and genetic approaches reveal that low-light-induced disease susceptibility is related to cellular oxidative stress in tomato

**DOI:** 10.1093/hr/uhad173

**Published:** 2023-08-29

**Authors:** Qian Luo, Jiao Wang, Ping Wang, Xiao Liang, Jianxin Li, Changqi Wu, Hanmo Fang, Shuting Ding, Shujun Shao, Kai Shi

**Affiliations:** Department of Horticulture, College of Agriculture and Biotechnology, Zhejiang University, Hangzhou 310058, China; Department of Horticulture, College of Agriculture and Biotechnology, Zhejiang University, Hangzhou 310058, China; Department of Horticulture, College of Agriculture and Biotechnology, Zhejiang University, Hangzhou 310058, China; Department of Horticulture, College of Agriculture and Biotechnology, Zhejiang University, Hangzhou 310058, China; Department of Horticulture, College of Agriculture and Biotechnology, Zhejiang University, Hangzhou 310058, China; Department of Horticulture, College of Agriculture and Biotechnology, Zhejiang University, Hangzhou 310058, China; Department of Horticulture, College of Agriculture and Biotechnology, Zhejiang University, Hangzhou 310058, China; Department of Horticulture, College of Agriculture and Biotechnology, Zhejiang University, Hangzhou 310058, China; Department of Horticulture, College of Agriculture and Biotechnology, Zhejiang University, Hangzhou 310058, China; Department of Horticulture, College of Agriculture and Biotechnology, Zhejiang University, Hangzhou 310058, China

## Abstract

The impact of low light intensities on plant disease outbreaks represents a major challenge for global crop security, as it frequently results in significant yield losses. However, the underlying mechanisms of the effect of low light on plant defense are still poorly understood. Here, using an RNA-seq approach, we found that the susceptibility of tomato to *Pseudomonas syringae* pv. *tomato* DC3000 (*Pst* DC3000) under low light was associated with the oxidation–reduction process. Low light conditions exacerbated *Pst* DC3000-induced reactive oxygen species (ROS) accumulation and protein oxidation. Analysis of gene expression and enzyme activity of ascorbate peroxidase 2 (APX2) and other antioxidant enzymes revealed that these defense responses were significantly induced by *Pst* DC3000 inoculation under normal light, whereas these genes and their associated enzyme activities were not responsive to pathogen inoculation under low light. Additionally, the reduced ascorbate to dehydroascorbate (AsA/DHA) ratio was lower under low light compared with normal light conditions upon *Pst* DC3000 inoculation. Furthermore, the *apx2* mutants generated by a CRISPR-Cas9 gene-editing approach were more susceptible to *Pst* DC3000 under low light conditions. Notably, this increased susceptibility could be significantly reduced by exogenous AsA treatment. Collectively, our findings suggest that low-light-induced disease susceptibility is associated with increased cellular oxidative stress in tomato plants. This study sheds light on the intricate relationship between light conditions, oxidative stress, and plant defense responses, and may pave the way for improved crop protection strategies in low light environments.

## Introduction

In nature, plant must be able to modify a range of biological processes to adapt to continually changing light conditions and other environmental circumstances. Light functions in the development of the plant due to photosynthetic carbon fixation [1], and is also pivotal in the dissemination of plant disease epidemics [2–4]. Plant disease outbreaks are more prone to develop in cloudy, foggy weather or other low light conditions [5, 6], resulting in a large crop loss and posing a significant risk to crop security worldwide. However, previous studies have predominantly focused on investigating the impacts of light quality and photoreceptors on plant immunity [7, 8]. The underlying molecular mechanisms of light intensity-regulated disease resistance have not been well elucidated.

The variations in light intensity levels mainly influence the light energy and photosynthetic flux, which are pivotal in the defense of plants against diseases. Photosynthesis not only modulates the energy status of the plant, influencing the ‘fuel’ availability required for launching defense responses, but also functions in the production of reactive oxygen species (ROS) related to defensive signaling [9, 10]. ROS include the superoxide radical (O_2_˙^−^), hydroxyl radical (OH˙), hydrogen peroxide (H_2_O_2_), and singlet oxygen (^1^O_2_) [11–14]. ROS can function as a signal to regulate an extensive array of abiotic stresses and pathogen defense [15–17]. Under high light intensity conditions, the surplus excitation energy induces various defense responses, including the bursts of ROS linked to enhanced resistance to virulent biotrophic bacteria [3, 18, 19]. Thus, a growing corpus of studies suggests that the ROS burst is pivotal in high light stimuli. Research findings indicate that LHCB5, a light-harvesting complex II protein of rice, is phosphorylated under high light conditions, thereby boosting plant defense against blast fungus *Magnaporthe oryzae* through activating the ROS burst in the chloroplasts [20]. Similarly, LHCB3 is also crucial for plant immunity, as *NbLHCB3* enhances ROS generation to defend against turnip mosaic virus (TuMV) [21]. The expression of *ascorbate peroxidase 2* (*APX2*) gene can be rapidly induced by high light stress, stimulating ROS generation in response to light changes in *Arabidopsis* [22]. Similarly, plants lacking AtRbohD demonstrated an impaired ability to upregulate the transcript levels of *catalase 1* (*CAT1*) and *APX1* under high light, implying that ROS could serve as an amplifier for signaling purposes [23].

In contrast to the well-established connection between high-light-induced ROS signaling and plant defense [20, 21, 24], the involvement of ROS in the defense responses under low light conditions has gained less attention. As high light induces an ROS burst as a signal, it is reasonable to hypothesize that the decrease in ROS may be responsible for the increased disease susceptibility of plants under low light. Described as being involved in ‘signaling’ or ‘oxidative stress’, ROS play a controversial role in physiological processes [13, 25]. Unexpectedly, when exposed to low light intensity, O_2_˙^−^ and H_2_O_2_ considerably increased and accelerated photoinhibition through direct oxidative damage to PSII in tomato leaves [6, 26]. Similarly, exposure to low light conditions led to elevated levels of ROS, which subsequently caused a cascade of oxidative injuries, including lipid peroxidation [27]. How ROS are involved in the regulation of plant defense under low light intensity remains unknown.

The photophilic tomato plant is an economically valuable fruit vegetable worldwide. However, abiotic stressors and pathogen infections can affect a large number of tomato cultivars. Actually, a devastating disease in greenhouses is *Pseudomonas syringae* pv. *tomato* DC3000 (*Pst* DC3000), which causes tomato bacterial speck disease. Outbreaks often occur in low light conditions, resulting in severe economic losses [28]. Here, combining transcriptomic and genetic methods, we found that susceptibility to *Pst* DC3000 in tomato was closely linked to cellular oxidative stress under low light conditions and exogenous ascorbate (AsA) treatment could reduce the increased susceptibility of *apx2* mutants to *Pst* DC3000. Our findings demonstrate that low-light-induced disease susceptibility is related to cellular oxidative stress, which can be helpful in elucidating the mechanism of disease resistance changes in plants under low light.

## Results

### Susceptibility of tomato to *Pst* DC3000 under low light is associated with the oxidation–reduction process

**Figure 1:**
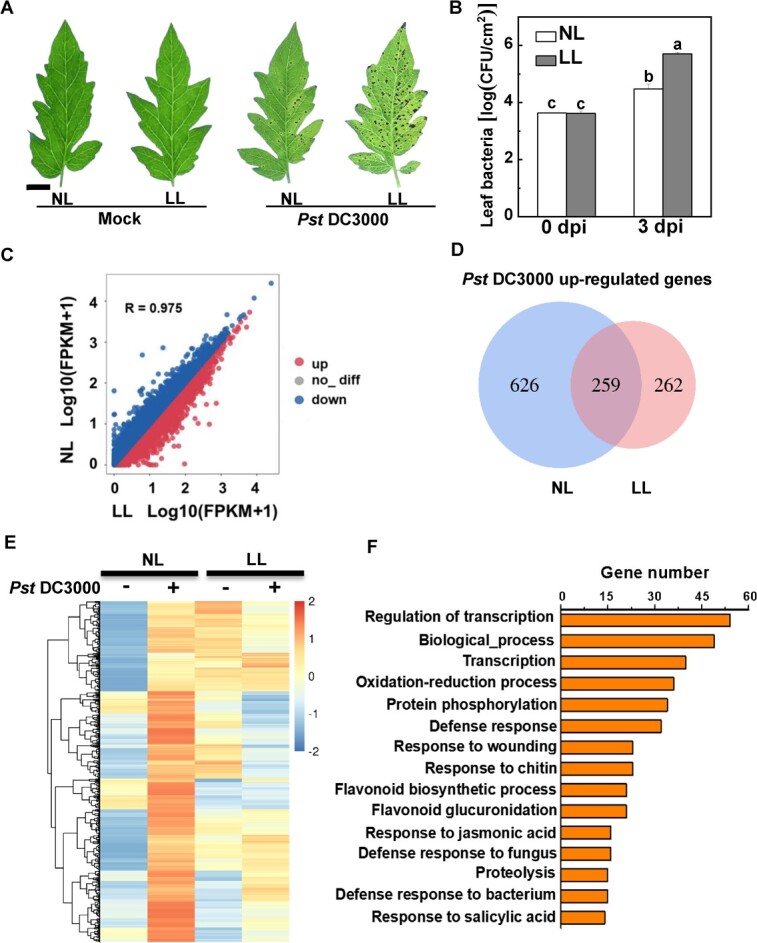
Susceptibility of tomato to *Pst* DC3000 under low light is associated with the oxidation–reduction process. Tomato plants were inoculated with *Pst* DC3000 under normal light intensity (NL, 300 μmol m^−2^ s^−1^) or low light intensity (LL, 50 μmol m^−2^ s^−1^). (A) Representative disease symptoms photographed 3 days post-inoculation (dpi). Scale bar, 1 cm. (B) Bacterial growth in tomato leaves at 3 dpi. Data are presented as mean values ± standard deviation, *n* = 3. Different letters indicate significant differences at *P* < .05 (Tukey’s test) among treatments. (C) Scatter plots of tomato whole-genome transcript fragments per kilobase of transcript per million mapped reads (FPKM) in LL vs NL with mock inoculation. Leaves were sampled 12 h post-inoculation (hpi). (D) Venn diagram showing the number of *Pst* DC3000-induced upregulated genes (fold change ≥2 and *P* < .05) in tomato plants under different light intensity conditions. The list of *Pst* DC3000-induced upregulated genes is shown in Supplementary Data Table S2. (E) Heat map depicting the *Pst* DC3000-induced fold change in transcript levels of 626 normal light-dependent genes in tomato plants. The list of normal light-dependent *Pst* DC3000-induced upregulated genes is shown in Supplementary Data Table S3. (F) Enriched GO biological process categories of normal light-dependent *Pst* DC3000-induced upregulated genes. The cluster gene number is ≥14. The lists are shown in Supplementary Data Table S4.

To examine the effects of light intensity on tomato resistance to *Pst* DC3000, tomato plants were subjected to either the normal light intensity (300 μmol m^−2^ s^−1^) or low light intensity (50 μmol m^−2^ s^−1^) with the same light quality, instantly after mock or *Pst* DC3000 inoculation. Low light intensity considerably increased more severe disease lesions and the bacterial population in the leaves than normal light intensity (Fig. 1A and B). To further investigate why the plant immune responses were decreased under low light, we performed RNA-seq analysis. The nearly linear correlation coefficient (*R*) (0.975) of all transcript expression levels between normal and low light showed that low light did not largely change the global transcript profile under mock-inoculated conditions (Fig. 1C). Following *Pst* DC3000 inoculation, more transcripts were altered in abundance (fold change ≥2, *P* < .05) under normal light compared with the low light condition. Following *Pst* DC3000 inoculation, the transcription levels of an additional 626 genes were substantially increased under normal light compared with their transcription under low light (Fig. 1D). Therefore, the 626 transcripts were identified as normal light-dependent *Pst* DC3000-induced genes. As shown by the heat map, a global decrease in the expression of *Pst* DC3000-induced genes was observed in low light conditions compared with the expression levels observed under normal light, following inoculation with the pathogen (Fig. 1E). Thirty-six out of 570 Gene Ontology (GO) items in the biological processes category were connected to the oxidation–reduction process, according to GO enrichment analysis (Fig. 1F).

### *Pst* DC3000-induced ROS accumulation and protein oxidation are aggravated under low light

**Figure 2:**
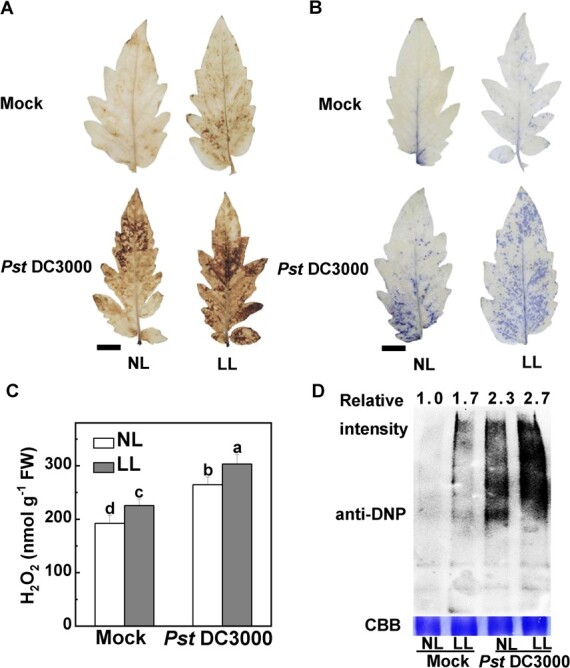
*Pst* DC3000-induced changes in ROS accumulation and protein oxidation under different light intensity conditions. Tomato plants were inoculated with *Pst* DC3000 under normal light intensity (NL, 300 μmol m^−2^ s^−1^) or low light intensity (LL, 50 μmol m^−2^ s^−1^). (A) Representative images of H_2_O_2_ accumulation as detected by DAB staining. Scale bar, 1 cm. (B) Representative images of O_2_˙^−^ accumulation as determined by NBT staining. Scale bar, 1 cm. (C) Quantification of H_2_O_2_. Data are presented as mean values ± standard deviation, *n* = 4. Different letters indicate significant differences at *P* < .05 (Tukey’s test) among treatments. (D) Levels of oxidized protein as detected by immunoblot analysis with anti-DNP antibody. All treated leaves above were sampled at 24 hpi.

To determine the involvement of the oxidation–reduction process in low-light-induced susceptibility, we evaluated the content of H_2_O_2_ and O_2_˙^−^ using DAB staining and NBT staining, respectively (Fig. 2A and B). Following *Pst* DC3000 inoculation, the tomato leaves subjected to low light showed an increase in brown and blue staining, indicating high levels of contents of H_2_O_2_ and O_2_˙^−^ respectively (Fig. 2A and B). Quantification of leaf H_2_O_2_ levels indicated that under low light H_2_O_2_ contents were higher than those under normal light, which was consistent with the histochemical staining (Fig. 2C). To determine the effects of low light on protein properties after *Pst* DC3000 inoculation, 2,4-dinitrophenol (DNP) and anti-DNP antibodies were used to evaluate the oxidation level of proteins in varying light intensities after *Pst* DC3000 inoculation. As shown in Fig. 2D, low light increased the level of protein oxidation more than normal light after *Pst* DC3000 inoculation. These findings suggest that the *Pst* DC3000-induced modulation of cellular redox homeostasis is impaired under low light.

### The antioxidant system is severely damaged under low light after *Pst* DC3000 inoculation

**Figure 3:**
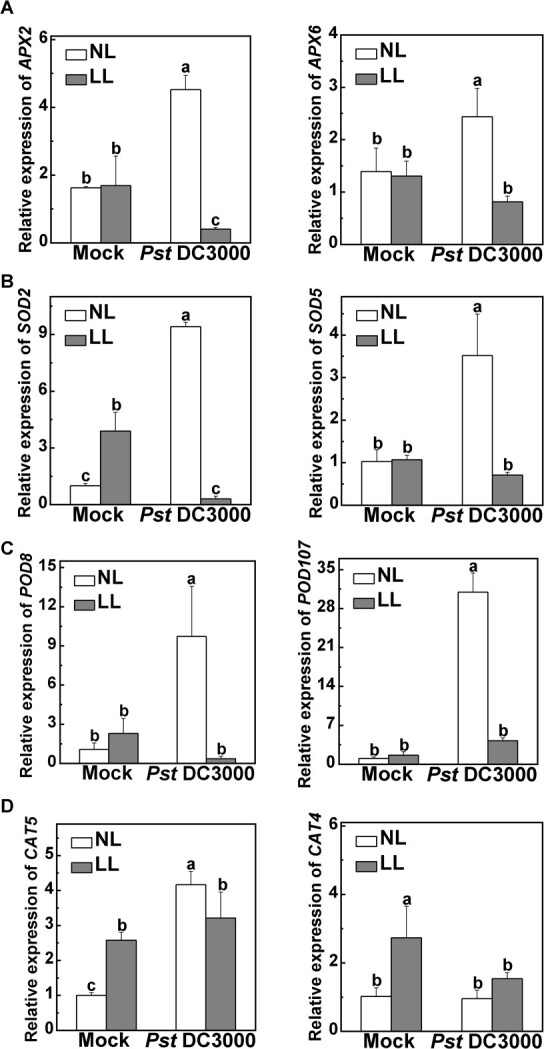
Changes in the transcript levels of tomato antioxidant enzyme family-related genes in response to combinations of pathogen and light treatments. Tomato plants were inoculated with *Pst* DC3000 under normal light intensity (NL, 300 μmol m^−2^ s^−1^) or low light intensity (LL, 50 μmol m^−2^ s^−1^). Relative expression of *APX2* and *APX6* (A), *SOD2* and *SOD5* (B), *POD8* and *POD107* (C), and *CAT5* and *CAT4* (D) in tomato plants leaves at 12 hpi. Data are presented as mean values ± standard deviation, *n* = 4. Different letters indicate significant differences at *P* < .05 (Tukey’s test) among treatments.

We further investigated antioxidant enzyme activities and antioxidant substance contents. The antioxidant enzymes, including ascorbate peroxidase (APX), superoxide dismutase (SOD), peroxidase (POD), and catalase (CAT), play critical roles in the defense against oxidative stress [12, 13]. The non-enzymatic antioxidants include AsA, glutathione and other metabolites, which maintain a steady state of redox homeostasis [16, 29]. These two antioxidative defense systems work together to maintain the balance between ROS production and scavenging in plants. We found that following *Pst* DC3000 inoculation many antioxidant enzyme-related genes were significantly upregulated under normal light but downregulated under low light, through analyzing the RNA-seq data (Supplementary Data Table S5). Then, the levels of antioxidant enzyme family-related gene expression were analyzed at 12 h post-inoculation (hpi) via qRT–PCR. The expression of *APX2, APX6*, *SOD2*, *SOD5*, *POD8*, *POD107*, and *CAT5* was significantly induced by *Pst* DC3000 inoculation under normal light, whereas their expression was not induced by the pathogen under low light (Fig. 3). Notably, following *Pst* DC3000 inoculation the expression levels of *APX2* and *SOD2* were even reduced under low light (Fig. 3A and B). Among them, the effects of low light combined with *Pst* DC3000 inoculation on *CAT* expression were less evident. After *Pst* DC3000 inoculation, the expression levels of *CAT5* and *CAT4* under low light were decreased less or nearly unchanged compared with their expression under normal light (Fig. 3D).

**Figure 4:**
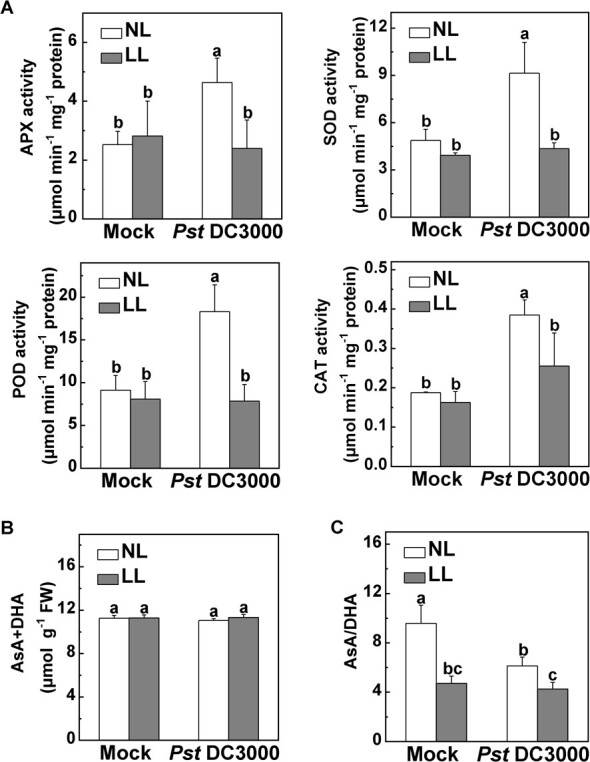
Regulation of cellular redox homeostasis in response to different combinations of pathogen and light treatment. Tomato plants were inoculated with *Pst* DC3000 under normal light intensity (NL, 300 μmol m^−2^ s^−1^) or low light intensity (LL, 50 μmol m^−2^ s^−1^). (A) Effects of different combinations of pathogen and light treatments on antioxidant enzyme activities, (B) total ascorbate content, and (C) redox status of ascorbate. AsA, reduced ascorbate; DHA, dehydroascorbate. Leaves were sampled at 24 hpi. Data are presented as mean values ± standard deviation, *n* = 4. Different letters indicate significant differences at *P* < .05 (Tukey’s test) among treatments.

In agreement with the results obtained from the RNA-seq and qRT–PCR analyses, the activities of the four antioxidant enzymes were significantly increased following pathogen treatment under normal light, while their activities were largely unchanged under low light (Fig. 4A). Similar to the transcriptional levels, the CAT activity showed a slight decrease under low light in contrast to that under normal light after *Pst* DC3000 inoculation (Fig. 4A). Additionally, as an abundant and stable antioxidant, AsA acts as a key role in the cytosol redox hub, and shifts in the ratio of reduced ascorbate to dehydroascorbate (AsA/DHA) are widely recognized as an index of redox status [30]. The ascorbate pool contents (including both AsA and DHA) were comparable among all treatments, regardless of *Pst* DC3000 inoculation or light intensity (Fig. 4B). However, under low light, the AsA/DHA ratios were reduced compared with those observed under normal light in both mock and *Pst* DC3000 inoculation treatments (Fig. 4C). In general, low light could significantly reduce antioxidant enzymes activities and AsA/DHA ratios in plants following *Pst* DC3000 inoculation to aggravate the oxidative stress.

### APX2 positively regulates disease resistance under low light

**Figure 5:**
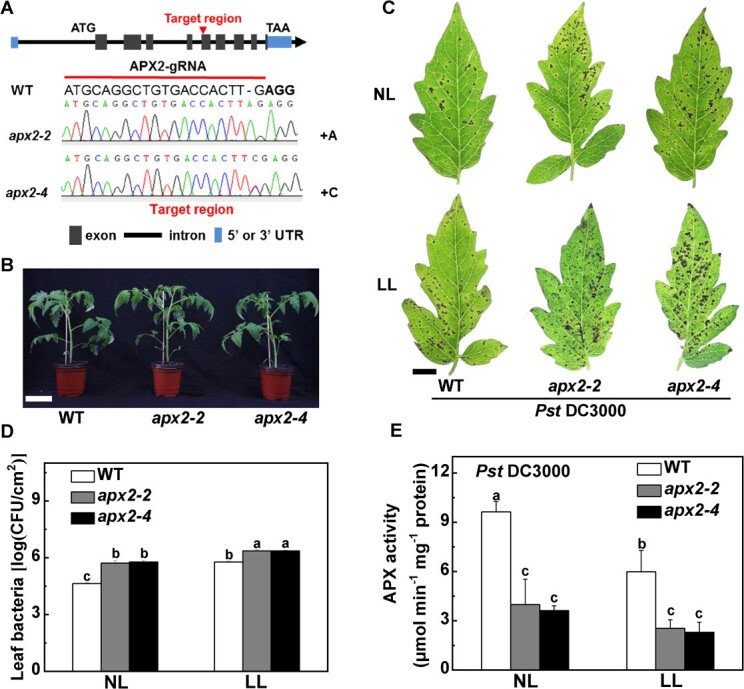
Role of APX2 in defense against *Pst* DC3000 under different light conditions. WT and *apx2* mutant tomato plants were inoculated with *Pst* DC3000 under normal light intensity (NL, 300 μmol m^−2^ s^−1^) or low light intensity (LL, 50 μmol m^−2^ s^−1^). (A) Schematic illustration of the sgRNA target site in WT and the missense mutations presented in two *apx2* alleles (*apx2-2* and *apx2-4*) from CRISPR-Cas9 *T*_2_ mutant lines. (B) Growth phenotype of *apx2* mutants. Photographs were taken at 40 days after sowing. Scale bar, 10 cm. (C) Representative disease symptoms of *apx2* mutants at 3 dpi under the two light intensity conditions. Scale bar, 1 cm. (D) Bacterial growth in *apx2* lines and WT plants at 3 dpi. (E) Effects of combinations of *Pst* DC3000 and light treatments on APX activities in mutants and WT plants. Leaves in *apx2* lines and WT were sampled at 24 hpi. Data in (D) and (E) are presented as mean values ± standard deviation, *n* = 4. Different letters indicate significant differences at *P* < .05 (Tukey’s test) among treatments.

Since there was a notable decrease in both *APX2* expression and APX activity under low light, we further investigated the contribution of APX2 in defense responses under varying light intensity conditions. Two independent tomato lines, *apx2-2* and *apx2-4*, with 1 bp of A and 1 bp of C insertion respectively, were generated and isolated from *T*_2_ progeny (Fig. 5A). The two *apx2* lines showed growth phenotypes similar to that of wild type (WT) (Fig. 5B). The *apx2* mutants showed reduced defense compared with the WT plants with regard to disease symptoms and bacterial growth, particularly under normal light conditions (Fig. 5C and D). The *apx2* mutants subjected to normal light conditions showed increased the bacterial numbers in the leaves by 23.2–24.4%, while the increase was only 10.1% when exposed to low light conditions (Fig. 5D). Moreover, the APX activities of the *apx2* mutants were lower compared with the WT plants following *Pst* DC3000 inoculation. Compared with normal light, the APX activities were reduced less under low light in both *apx2* lines (Fig. 5E). These results indicate that APX2 positively regulates defense under both light intensities, and the low-light-decreased APX activity may be the main reason for the disease susceptibility under low light conditions.

### Susceptibility of *apx2* mutants to *Pst* DC3000 under low light is alleviated by exogenous ascorbate

**Figure 6:**
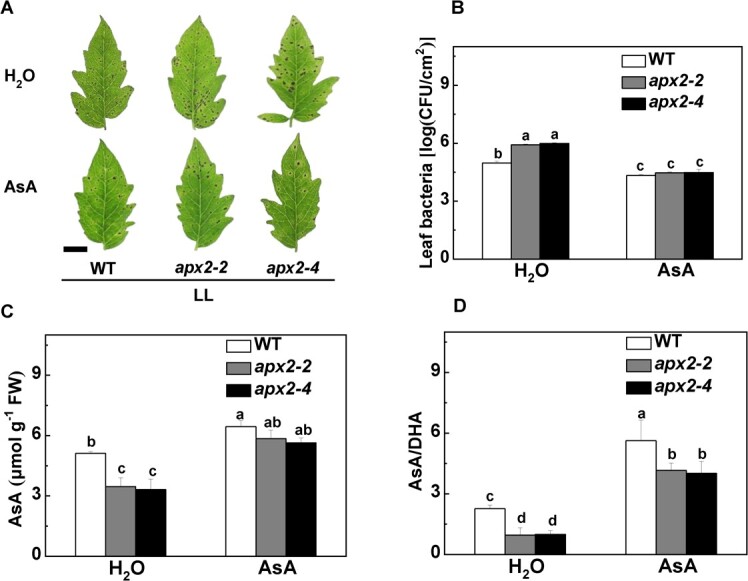
Susceptibility of *apx2* mutants to *Pst* DC3000 under low light is alleviated by exogenous AsA. WT and *apx2* mutant tomato plants were exogenously sprayed with 10 mM AsA or H_2_O for 3 days, and then were inoculated with *Pst* DC3000 under low light intensity (LL, 50 μmol m^−2^ s^−1^). (A) Representative disease symptoms photographed at 3 dpi under low light conditions as influenced by AsA. Scale bar, 2 cm. (B) Bacterial growth in leaves of *apx2* mutants and WT plant at 3 dpi with or without AsA pretreatment. (C, D) Effects of exogenous AsA on the endogenous AsA content (C) and AsA/DHA ratios (D) in *apx2* mutants and WT plants at 24 hpi with low-light treatment. Data in (B), (C), and (D) are presented as mean values ± standard deviation, *n* = 4. Different letters indicate significant differences at *P* < .05 (Tukey’s test) among treatments.

We further clarified whether exogenous AsA treatment could reduce the susceptibility of *apx2* mutants to *Pst* DC3000 exposed to low light. As predicted, exogenous AsA application enhanced disease resistance in both the *apx2* mutants and WT plants (Fig. 6A). Additionally, under low light conditions, AsA was able to alleviate the susceptibility of *apx2* mutants to *Pst* DC3000 to a level comparable to that of the WT plants (Fig. 6B). Meanwhile, exogenous AsA application enhanced endogenous AsA contents as well as the AsA/DHA ratios of all lines when exposed to low light, especially in the *apx2* mutants, leading to enhanced defense (Fig. 6C and D). Therefore, under low light, ascorbate metabolism appears to be crucial for the plant’s resistance to *Pst* DC3000.

## Discussion

In nature, plants have to withstand a diverse array of challenges that occur constantly, including enduring stressors like light stress and pathogenic assaults. Based on extensive prior research, it has been established that photoreceptors and high light intensity are intricately associated with the mechanisms governing plant defense against pathogens [2, 4, 31]. However, the underlying mechanisms of low light stress on plant defense remain poorly elucidated. Several lines of evidence are presented in this work to support the conclusion that, when exposed to low light, the susceptibility to *Pst* DC3000 is closely related to oxidative stress in tomato plants, which is due to the damage of cellular redox homeostasis.

An important inquiry revolves around the involvement of ROS signaling or oxidative stress in the susceptibility of plants to pathogens when exposed to low light intensity. Here, we found that, when exposed to low light conditions, alterations in plant pathogen resistance are associated with oxidative stress. Analysis of the transcriptome revealed that the susceptibility of tomato to *Pst* DC3000 under low light is related to the oxidation–reduction process (Fig. 1F). Moreover, the data presented here show that either *Pst* DC3000 inoculation or low light led to oxidative stress in tomato plants, which was aggravated in combined treatment with low light and *Pst* DC3000 (Fig. 2). In another, similar study combining salt stress and *P. syringae* inoculation, the oxidative burden was considerably amplified compared with the separate impacts of biotic stress [32]. However, it is commonly documented that apoplast-derived ROS from *Pst* DC3000 inoculation usually serve as positive plant immune signaling [33]. Like most abiotic stresses, varying levels of light intensity stress typically result in the buildup of ROS [34, 35]. The considerable increase in O_2_˙^−^ and H_2_O_2_ under low light conditions causes oxidative stress [6]. Therefore, we speculate that the repercussions of low-light-driven ROS extend far beyond the signaling functions of ROS induced by *Pst* DC3000 inoculation, ultimately causing progressive oxidative stress.

To counteract the overproduced ROS during diverse environmental stresses, an effective antioxidative system is employed, comprising both enzymatic antioxidants and non-enzymatic AsA–GSH cycles [36, 37]. The functions of specific antioxidant enzymes and non-enzymatic antioxidants within the antioxidative system have been elucidated in previous research, shedding light on their involvement in light stress responses and interactions between plants and pathogens. For example, an elevation in antioxidant enzymes activities, including APX, SOD, and CAT, has been observed in *Festuca arundinacea* plants when transferred to high light from low light conditions [38]. *Arabidopsis cat2* mutants show a constitutive upregulation of pathogen response proteins [39, 40]. The expression pattern of the antioxidative system response to the production of ROS induced by pathogen infection combined with low light stress remains unclear. Here, we found that low light inhibited the gene expression and activity of antioxidant enzymes and AsA/DHA ratios following *Pst* DC3000 inoculation in tomato (Figs 3 and 4A). Similarly, a previous study documented that separate *P. syringae* pv. *lachrymans* (*Psl*) inoculation significantly increased MnSOD and CuZnSOD, but following salt pretreatment 2 days after *Psl* inoculation there was a reduction in the total SOD activity compared with plants inoculated separately with *Psl* [32]. The observations from these findings indicate that the antioxidant system undergoes significant damage with pathogen infection combined with low-light stress. Therefore, when exposed to low light, excessive ROS cannot be effectively cleared, eventually resulting in oxidative stress in tomato with *Pst* DC3000 inoculation.

Remarkably, *APX2* gene expression was found to be diminished even more when subjected to low light conditions in combination with *Pst* DC3000 inoculation (Fig. 3A). Actually, we found that APX2 positively regulates disease resistance in different light intensity conditions, especially under low light. The *apx2* mutation led to increased disease and reduced APX activity compared with the WT plants in both light intensities, particularly under normal light conditions (Fig. 5C and D). The application of exogenous ASA confirmed this conclusion. Indeed, exposed to low light conditions, the endogenous AsA content, along with the AsA/DHA ratio, exhibited an increase in all lines when exogenously applied with AsA, especially in the *apx2* mutants (Fig. 6C and D). Thus, exogenous AsA alleviates oxidative stress in leaves by increasing endogenous ASA accumulation [41–44]. The regulatory mechanisms governing *APX* gene expression and cytosolic APX isoform activity are influenced by a range of stressors promoting ROS generation. These stressors include but are not limited to high light, exposure to heavy metals, pathogenic assaults, and other, similar factors [22, 45–47]. An example to illustrate this is the plants’ enhanced resistance to cooling and high light stresses, achieved through the overexpression of the *tAPX* gene [48]. Overall, in the plant, *APX2* seems to be a crucial gene in multiple stresses by modulating the antioxidant system.

### Conclusion

The findings presented in this study reveal a strong association between oxidative stress and the increased susceptibility of tomato to *Pst* DC3000 under low light. This study emphasizes the significance of cellular redox homeostasis in plant resistance under low light.

## Materials and methods

### Plant material and growth conditions

The tomato (*Solanum lycopersicum* L.) variety Condine Red (CR) was used as WT in the present study. CRISPR/Cas9 gene-editing-mediated stable *apx2* mutants were generated as described previously [47]. Tomato seeds were germinated in a sterile 1:3 (v/v) mixture of vermiculite and peat containing growth substrates, receiving daily Hoagland nutrient solution. Approximately 2 weeks later, tomato seedlings with two fully expanded true leaves were transferred to plastic pots. The growth conditions were as follows: 22°C/20°C (day/night) air temperature, 12 h daily photoperiod, 400 μmol m^−2^ s^−1^ photosynthetic photon flux density (PPFD), 75% relative humidity. Plants ~5 weeks old were used for each treatment.

### Light, bacterial, and ascorbate treatments

For *in planta* light and bacterial combination treatment, tomato CRISPR/Cas 9-mediated lines of *apx2* and WT plants exposed to low light (LL, 50 μmol m^−2^ s^−1^) or control normal light (NL, 300 μmol m^−2^ s^−1^) were simultaneously subjected to *P. syringae* inoculation. Other environmental conditions except light were the same as above. After 24 h combination treatments, pooled samples were collected for the measurement of ROS-related parameters. The leaves of *apx2* mutants and WT plants received three daily applications of 10 mM AsA or H_2_O as control before light and *P. syringae* inoculation treatments. The representative plant images were taken after 3 days of different light, *P. syringae*, and AsA treatments.

### Pathogen inoculation and bacterial growth analysis

The bacterium *Pst* DC3000 was cultured at 28°C overnight in King’s B medium supplemented with rifampicin (25 mg ml^−1^), and diluted next day with 10 mM MgCl_2_. Tomato plants were inoculated with the bacterial suspension at a final concentration of 10^7^ colony-forming units (CFU) ml^−1^ with 0.02% Silwet L-77 [49]. MgCl_2_ buffer with 0.02% Silwet L-77 was applied as a mock inoculation. After 3 days of pathogen inoculation, the bacterial population counts (CFU) were measured from four leaves per plant in accordance with previous approaches [50].

### ROS analysis

To assess ROS accumulation, tomato leaves were stained with 1 mg ml^−1^ DAB in 50 mM Tris–HCl (pH 3.8) for 24 h to detect H_2_O_2_ levels or 0.1 mg ml^−1^ NBT in 25 mM HEPES buffer (pH 7.8) for 2 h to detect O_2_˙^−^ levels in the dark [47]. The levels of H_2_O_2_ in tomato leaves were estimated by measuring absorbance at 412 nm.

### Immunoblotting assay

An OxyBlot Protein Oxidation Detection Kit (Chemicon International, Temecula, CA, USA) was used to evaluate the oxidized protein that was extracted from the soluble protein according to the manufacturer’s instructions.

### Measurements of antioxidant contents and enzyme activities

To measure the antioxidant contents, including ascorbate and glutathione assays, ~0.1 g of leaf tissue was pulverized into a fine powder using liquid nitrogen. The powdered tissue was then extracted using 1 ml of 0.2 M HCl. The subsequent neutralization of supernatant, detection, and calculation of ascorbate and glutathione contents were performed according to a previously defined procedure [51].

To measure antioxidant enzyme activity, the supernatants for enzyme activity analysis were extracted from 0.3 g leaves with 3 ml buffer containing 25 mM HEPES, 0.2 mM EDTA, 2 mM AsA, and 2% polyvinylpolypyrrolidone (w/v) (pH 7.8). The enzyme activities of APX and CAT were determined according to Hu *et al*. [47], and SOD and POD were determined according to Cheng *et al*. [52].

### Gene expression analysis

Total RNA was isolated from plant tissues using an RNA extraction kit (TIANGEN, Zhejiang, China) and reverse transcribed using a ReverTra Ace qPCR–RT Kit (Toyobo, Tokyo, Japan). Quantitative real-time PCR (qRT–PCR) was performed on optical 384-well plates in the QuantStudio5 instrument (Thermo Fisher Scientific, MA, USA) with 10 μl reaction buffer containing 5 μl SYBR SuperMix (Vazyme Biotech, Nanjing, China), 3.6 μl water, 1 μl cDNA, and 0.2 μl each of forward and reverse primers. The primers used for the target genes and the internal control *ACTIN* gene are listed in Supplementary Data Table S1.

### RNA-seq library preparation and sequencing

Tomato leaves were taken at 12 h after *Pst* DC3000 infection and low light treatment, and instantly frozen in liquid nitrogen to extract RNA for sequencing. RNA-seq was conducted at LC-Bio Technologies (Hangzhou, China). The red dots represent upregulated (fold change ≥ 2 and P < 0.05) genes, whereas the blue dots represent downregulated (fold change ≥ 2 and P < 0.05) genes in scatter plots of tomato whole-genome transcript FPKM.

### Statistical analysis

At least three independent biological replicates sampled from different plants were included in each experiment. The experiments were independently performed two or three times. The differences among treatment means were determined via SAS 8.0 software (SAS Institute), and the averages were compared using Tukey’s test at the 5% level.

## Acknowledgements

This work was supported by the National Natural Science Foundation of China (32172650) and the Key Research and Development Program of Zhejiang Province (2021C02040).

## Author contributions

K.S. conceived the research; Q.L., J.W., and K.S. designed the experiments; Q.L., P.W., X.L., J.L., C.W., H.F., S.D., and S.S. performed the research and analyzed the data; and Q.L., J.W., and K.S. wrote the article with contributions from other authors.

## Data availability

The data used to support the findings of this study are included within the article and the supporting information file. RNA-seq datasets can be accessed in the SRA database (https://www.ncbi.nlm.nih.gov/sra/PRJNA990324) under record number PRJNA990324. Sequence data from this article can be found in the Sol Genomics Network (http://solgenomics.net/) under the following accession numbers: *APX2*, Solyc06g005150, *APX6*, Solyc11g018550, *SOD2*, Solyc02g021140, *SOD5*, Solyc06g048410, *POD8*, Solyc07g042440, *POD107*, Solyc04g071900, *CAT5*, Solyc12g094620, *CAT4*, Solyc04g082460, *SlACTIN,* Solyc03g078400.

## Conflict of interests

The authors declare that they have no conflict of interest.

## Supplementary information

Supplementary data is available at *Horticulture Research* online.

## Supplementary Material

Web_Material_uhad173
